# Effects of Fremanezumab on Psychiatric Comorbidities in Difficult-to-Treat Patients with Chronic Migraine: Post Hoc Analysis of a Prospective, Multicenter, Real-World Greek Registry

**DOI:** 10.3390/jcm12134526

**Published:** 2023-07-06

**Authors:** Michail Vikelis, Emmanouil V. Dermitzakis, Georgia Xiromerisiou, Dimitrios Rallis, Panagiotis Soldatos, Pantelis Litsardopoulos, Dimitrios Rikos, Andreas A. Argyriou

**Affiliations:** 1 Headache Clinic, Mediterraneo Hospital, 16675 Athens, Greece; 2 Euromedica General Clinic, 54645 Thessaloniki, Greece; manolis.dermitzakis@gmail.com; 3 Department of Neurology, University Hospital of Larissa, University of Thessaly, 41110 Larissa, Greece; georgiaxiromerisiou@gmail.com; 4 Department of Neurology, Tzaneio General Hospital of Piraeus, 18536 Athens, Greece; jimrallis@hotmail.com; 5 Independent Researcher, 24100 Kalamata, Greece; soldatosp@gmail.com; 6 Headache Outpatient Clinic, Department of Neurology, Agios Andreas State General Hospital of Patras, 26335 Patras, Greece; pantelis84@hotmail.com (P.L.); andargyriou@yahoo.gr (A.A.A.); 7 404 Military Hospital, 41222 Larisa, Greece; rikosd@hotmail.com

**Keywords:** CGRP, monoclonal antibodies, chronic migraine, fremanezumab, psychiatric comorbidities, efficacy, response

## Abstract

Objective: this post hoc analysis aimed to evaluate the efficacy of fremanezumab in difficult-to-treat chronic migraine (CM) patients with and without psychiatric comorbidities (PCs), mainly anxiety and/or depression. Methods: We assessed data from CM patients with and without PCs who failed at least 3 preventives and eventually received at least 3 consecutive monthly doses of fremanezumab 225 mg. Outcomes included the crude response (≥50% reduction in monthly headache days (MHDs)) rates to fremanezumab from the baseline to the last clinical follow-up. The changes in MHDs; MHDs of moderate/greater severity; monthly days with intake of abortive medication; and the proportion of patients’ changing status from with PCs to decreased/without PCs were also compared. Disability and quality of life (QOL) outcomes were also assessed. Results: Of 107 patients enrolled, 65 (60.7%) had baseline PCs. The percentage of patients with (*n* = 38/65; 58.5%) and without (*n* = 28/42; 66.6%) PCs that achieved a ≥50% reduction in MHDs with fremanezumab was comparable (*p* = 0.41), whereas MHDs were significantly reduced (difference vs. baseline) in both patients with PCs (mean −8.9 (standard error: 6.8); *p* < 0.001) and without PCs (−9.8 (7.5); *p* < 0.001). Both groups experienced significant improvements in all other efficacy, disability, and QOL outcomes at comparable rates, including in MHD reduction. A significant proportion of fremanezumab-treated patients with baseline PCs de-escalated in corresponding severities or even reverted to no PCs (28/65; 43.1%) post-fremanezumab. Conclusions: fremanezumab appears to be effective as a preventive treatment in difficult-to-treat CM patients with and without PCs while also being beneficial in reducing the severity of comorbid anxiety and/or depression.

## 1. Introduction

Migraine ranks among the most common primary headache disorders. It is characterized by attacks of headache and associated symptoms, including nausea, vomiting, photophobia, or phonophobia. Most patients suffer from episodic migraine (EM) and experience migraine attacks on less than 15 days per month. However, up to 6% of individuals with EM progress to chronic migraine (CM), defined as headaches occurring on 15 or more days per month, with migrainous features or a response to migraine-specific medications for at least 8 days per month. Chronic migraine can be with or without aura, and although there is a requirement for symptoms to be present for three months in order to diagnose it, most patients experience it for years before being diagnosed. A sizable proportion of CM patients experience severe headaches almost daily, resulting in the generation of considerable disability, a deterioration in their quality of life, and evidence of clinically significant psychological distress [1].

Migraine and psychiatric disorders, mainly depression and/or anxiety, are frequently comorbid in CM, with a lifetime prevalence of depression significantly above the corresponding prevalence seen in controls without migraine [2] or even in EM patients [3]. Moreover, a bidirectional association between psychiatric comorbidities (PCs) and migraine has been identified, as the frequency of monthly headache days (MHDs) proportionally increases with the frequency and severity of PCs. Thus, the comorbid depression and/or anxiety significantly contributes to increasing the risk of progression from EM to CM [4].

A number of non-specific medications (e.g., beta blockers, anticonvulsants, and antidepressants) have long been used for migraine prevention; however, clinical experience has shown that both the efficacy and safety/tolerability of these medications is rather modest, and there is a need for optimized and specific migraine-preventive medications, rather than the current standard of care with the use of oral preventatives, such as topiramate [5,6]. The recognition of calcitonin gene-related peptide (CGRP) as a neuropeptide critically involved in both central and peripheral (neuronal, sensitization, vasodilation, inflammation, and protein extravasation) processes underlying the pathophysiology of migraine has revolutionized the prophylactic treatment of migraine [7]. Several clinical studies have shown that targeted therapies with the use of anti-CGRP monoclonal antibodies (anti-CGRP MAbs) were efficacious and safe in the prophylaxis of both EM and CM [8,9].

Fremanezumab is a fully humanized IgG 2 (delta) a/kappa monoclonal antibody that was approved in September 2018 in the United States and in January 2019 by the EMA/CHMP for the prophylactic treatment of both EM and CM, based on its ability to potently and selectively bind to both CGRP isoforms (α- and β-CGRP), preventing them from binding to the CGRP receptor in the trigeminal ganglion and meningeal nociceptors in order to selectively inhibit the activation of the trigeminovascular pain pathway [10,11,12]. Subsequently, the excellent efficacy/safety profile of fremanezumab in migraine prophylaxis was documented in several real-world studies [9,13].

Post hoc analyses of data from the HALO CM study demonstrated significant reductions in MHDs in patients with comorbid depression, significant reductions in disability, and significant improvements in QOL outcomes. Additionally, a significant proportion of patients with evidence of baseline major depressive symptomatology experienced 50% reductions in severities, as assessed by the PHQ-9 scale, over the study period while treated with fremanezumab, thus providing evidence for the possibility of improvements in both migraine and depression [14].

Fremanezumab received market authorization in Greece for migraine prevention in 2020 and was granted a positive opinion from the national insurance organization (EOPPY) in 2021 to be fully reimbursed in patients suffering from high-frequency EM (HFEM: 8–14 days/month) who previously failed to first-line treatments. CM reimbursement additionally requires failure to onabotulinumtoxinA. We have recently demonstrated in real-world conditions that fremanezumab was able to be effective and safe when administered for migraine prophylaxis in difficult-to-treat migraine patients with either HFEM or CM [15]. We subsequently reported that a precise phenotypic profiling with the identification of pain characteristics consistent with peripheral and/or central sensitization might reliably predict the responsiveness to fremanezumab [16]. In the latter post hoc analysis, the baseline occurrence of PCs was not found to be associated with response to fremanezumab, and patients responded equally regardless of comorbid PCs [16].

To specifically test the latter clinical scenario in a homogenous cohort of CM patients, the aim of this post hoc analysis of data extracted from a prospective, multicenter, Greek registry was to evaluate the efficacy of fremanezumab in difficult-to-treat CM patients with and without PCs, mainly anxiety and/or depression.

## 2. Materials and Methods

The study was conducted in accordance with the requirements of the Declaration of Helsinki, the protocol was approved by the Institutional Review Board of “Agios Andreas” Patras General Hospital, and each patient provided an informed consent before entering the study. In this post-hoc analysis of data extracted from a prospective, observational study, the study population was composed of male or female patients, aged 18 years and older, diagnosed with CM with or without medication-overuse headache (MOH), who were prescribed fremanezumab as a treatment decision of their physician before enrollment in this study. Fremanezumab treatment was commenced strictly in line with the approved indication as described in the Summary of Product Characteristics (SmPC) [17] and current national reimbursement policies. These policies dictate that full reimbursement of fremanezumab is granted in CM patients who inadequately responded or were intolerant to first-line oral treatments and onabotulinumtoxinA, given quarterly for 3 consecutive courses [18,19].

Eligibility was confirmed by a protocol-specific checklist. Adult patients were included in the study only if all of the following criteria were met: (i) the patient had read and signed the informed consent after receiving all information about the study; (ii) the patient had a formal diagnosis of CM, according to the international diagnostic criteria [20]; (iii) the patient has been prescribed fremanezumab as a treatment decision of their physician, according to the SmPC [17]; (iv) the patient was naïve to prior exposure with anti-CGRP MAbs; (v) the patient had been maintaining a daily headache diary as part of their routine disease management and had maintained the diary for at least 21 days in the 28 days prior to fremanezumab treatment initiation for 2 consecutive months; (vi) the patient’s headache diary ideally captured information on headache duration, headache severity, headache characteristics, and days with intake of any acute medication for headache relief; (vii) the patient was able to understand and was willing to keep records in the paper headache diary for the course of the study. Exclusion criteria included any contraindication to fremanezumab, according to the standard clinical practice and the approved SmPC [17]. Patients participating in any interventional clinical trial in CM, patients who were pregnant, or patients who were nursing females were excluded. Moreover, patients with major psychiatric disorder, such as autism, uncontrolled bipolar disorder, and schizophrenia, were also excluded from participating in this study.

Fremanezumab (Ajovy^®^ 225mg/pf-syr, Teva Pharma, Athens-Greece) was prescribed either as 225 mg monthly (every 28–30 days) or 675 mg quarterly (every 90 days), depending on the decision of the patient’s physician and standards of care. Patients were guided by their treating physician to use their fremanezumab solution for self-injection, as described in the SmPC, for at least 3 months (12 weeks) before establishing efficacy. The end of the observational period was defined as the last routine clinic visit during the observational period of each patient. Hence, patients in this group were followed for 3–18 months of fremanezumab exposure.

Effectiveness data were evaluated using the information recorded by patient-reported outcome measures in the patients’ diaries in paper format (headache diary compliance was set to at least 80% of total days) and from validated headache-related disability tools, including questionnaires, used in real-world clinical practice. The primary endpoint was to evaluate the mean change from baseline in the monthly average number of migraine days (MHDs) at the last routine follow-up, whereas the secondary endpoints for effectiveness were the following: (i) the proportion of patients reaching at least 50% reduction in the mean MHDs during the clinical follow-up period (>3 months) after the first dose of fremanezumab; (ii) the change in mean MHDs with peak moderate/severe headache intensity, i.e., more than 4 out of 10 on a 0–10 numerical scale; (iii) the change in mean monthly days with consumption of any abortive headache medications; (iv) the documentation of changes in disability score, as measured by the Migraine Disability Assessment (MIDAS) questionnaire [21], and also in the headache-related disability score, as measured by the 6-item headache impact test (HIT-6) [22] and quality of life assessment, as assessed by the EQ-5D questionnaire [23]. EQ-5D is composed of a “self-classifier” part and a thermometer-like vertical Visual Analog Scale (VAS), by which respondents can self-rate their perceived health status with a grade ranging from 0 to 100, with higher scores indicative of higher health status [23]. The incidence and severity of psychological distress at baseline to document PCs was assessed using the HADS [24] scale, consisting of 14 items: 7 for anxiety (HADS-A) and 7 for depression (HADS-D). All items are based solely on the psychological symptoms of mood and anxiety disorders to the exclusion of somatic symptoms. Each subscale is scored from 0 to 21, with scores of 0–7 representing a non-case of anxiety and depression, 8–10 a doubtful or borderline case, and 11–21 a definite case.

Finally, the patients’ perception of the impact of fremanezumab treatment on disease management and satisfaction was evaluated with the use of the 7-point (1 stands for “no change” and 7 for “considerable improvement”) self-report “Patient Global Impression of Change” (PGIC) questionnaire [25]. The cut-off score to define a “clinically significant benefit” was set to a PGIC score of ≥5, according to the IMMPACT recommendations [26].

### Statistical Analysis

Descriptive statistics were generated for all variables. Two-sided chi-squared tests were used to compare categorical data between patients with baseline PCs vs. those without PCs. For within-group comparisons, the paired-samples *t*-test was used to reveal any potential changes in mean headache outcome scores from baseline to post-fremanezumab follow-up. For between-group comparisons, the changes in mean headache outcome scores were evaluated by subtracting each patient’s baseline value from her/his last value and were calculated with the use of the independent-sample *t*-tests. Unless otherwise stated, all tests were two-sided, and significance was set at *p* < 0.05. Statistics were performed by employing the SPSS for Windows (release 27.0; SPSS Inc., Chicago, IL, USA).

## 3. Results

The study sample consisted of 14 males (13.1%) and 93 females (86.9%) with a mean age of 49.8 ± 10.7 (range: 23–70) years. Of 107 patients enrolled, 65 (60.7%) had baseline PCs. The majority of them (*n* = 37; 56.9%) had a normal BMI of <24.9 and were diagnosed with concurrent MOH (*n* = 61; 93.8%). Among PCs, mixed anxiety and depression disorders were most commonly seen (*n* = 26; 40%), followed by anxiety disorders (*n* = 24; 36.9%), depression (*n* = 13; 20%), and bipolar disorders (*n* = 2; 3.1%). These patients were treated with venlafaxine (*n* = 22), duloxetine (*n* = 10), amitriptyline (*n* = 24), and other SSRIs (*n* = 9). The overall baseline epidemiological and clinical characteristics of participants, according to whether they had PCs or not at baseline, are described in Table 1.

### 3.1. Within-Group Comparison of Fremanezumab-Related Efficacy Headache Outcomes, according to Baseline Evidence or Lack of Psychiatric Comorbidities

#### 3.1.1. Fremanezumab-Treated Patients without Baseline PCs (*n* = 42)

MHDs were significantly reduced (difference vs. baseline) in patients without PCs (22.4 ± 5.0 vs. 12.6 ± 7.6; *p* < 0.001). Likewise, there was a significant decrease in MHDs with moderate/severe headache (more than 4/10 in VAS) compared with the baseline (16.8 ± 6.5 vs. 9.7 ± 7.7; *p* < 0.001), whereas the number of monthly days with intake of acute headache medications was also significantly lower (18.9 ± 6.6 vs. 10.5 ± 7.3; *p* < 0.001).

A total of 28/42 (66.6%) patients had ≥50% reduction in MHDs with fremanezumab and were as such defined as treatment responders. Among them, 17 and 11 patients successfully achieved response at 50% and 75%, respectively, after treatment with fremanezumab. As expected, the efficacy to therapy influenced the disability and QOL outcomes. MIDAS (113.4 ± 69.3 vs. 54.6 ± 52.6; *p* = 0.018) and HIT-6 (67.4 ± 8.4 vs. 59.5 ± 10.5; *p* = 0.025) scores decreased, and the EQ5D scores (46.6 ± 19.3 vs. 68.5 ± 20.3; *p* = 0.05) increased. All 28 treatment responders in this group remained satisfied to score ≥5 on PGIC; specifically, 7 scored 5, 18 scored 6, and 3 scored 7 on PGIC.

#### 3.1.2. Fremanezumab-Treated Patients with Baseline PCs (*n* = 65)

Comparably to patients without PCs, participants with evidence of psychopathology experienced a significant decrease in MHDs between the baseline and last efficacy follow-up (23.9 ± 5.0 vs. 14.1 ± 7.8; *p* < 0.001). Likewise, there was a significant decrease in the number of MHDs with peak headache intensity of ≥5 (17.3 ± 5.1 vs. 10.4 ± 6.4; *p* < 0.001) and also in monthly days with intake of acute headache medications (21.8 ± 6.1 vs. 12.8 ± 7.9; *p* < 0.001). A total of 38/65 (58.5%) patients were classified as responders as they achieved a ≥50% decrease in MHDs with fremanezumab: 23 at 50% and 15 at 75%. The changes in disability and QOL outcomes clearly favored fremanezumab treatment, which was demonstrated by the reduced MIDAS (111.4 ± 58.7 vs. 67.5 ± 54.4; *p* = 0.002) and HIT-6 (70.0 ± 7.4 vs. 61.6 ± 11.2; *p* < 0.001) scores and a strong tendency of significantly higher EQ5D scores (45.9 ± 20.5 vs. 62.9 ± 22.7; *p* = 0.08). All 38 treatment responders in this group were satisfied with fremanezumab treatment and scored ≥5 on PGIC; specifically, 12 scored 5, 24 scored 6, and 2 scored 7 on PGIC. Finally, a significant proportion of fremanezumab-treated patients with baseline PCs de-escalated in corresponding severities or even reverted to no PCs (28/65; 43.1%) post-fremanezumab. In support of the latter finding, there were improvements in both HADS-A (13.4 ± 4.1 vs. 11.1 ± 4.1; *p* < 0.001) and HADS-D scores (11.9 ± 4.6 vs. 10.2 ± 3.5; *p* < 0.001) post-fremanezumab, compared with the baseline. The improvements in PC severities persisted throughout the study period.

### 3.2. Between-Group Comparison of Fremanezumab-Related Efficacy Headache Outcomes, according to Baseline Evidence or Lack of Psychiatric Comorbidities

**Figure 1:**
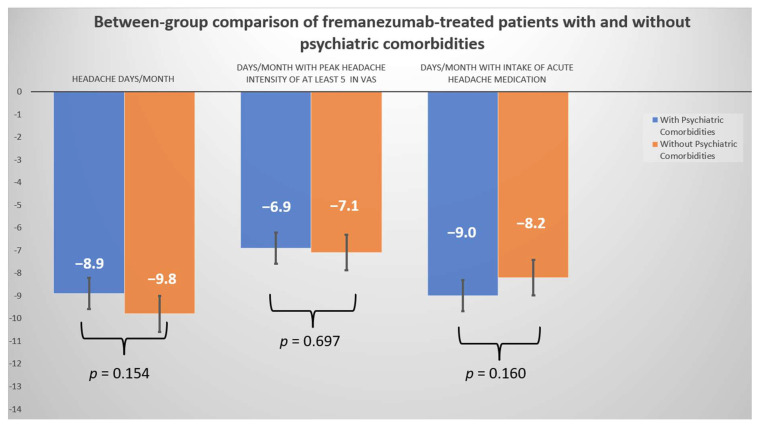
Changes in mean fremanezumab-related headache efficacy scores from baseline to the last follow-up between treatment groups.

Both groups experienced significant improvements in all efficacy, disability, and QOL outcomes at comparable rates, including in MHD reduction. Figure 1 shows the between-group changes in all fremanezumab-related efficacy headache outcomes, compared with the baseline, in CM patients with and without PCs.

**Figure 2:**
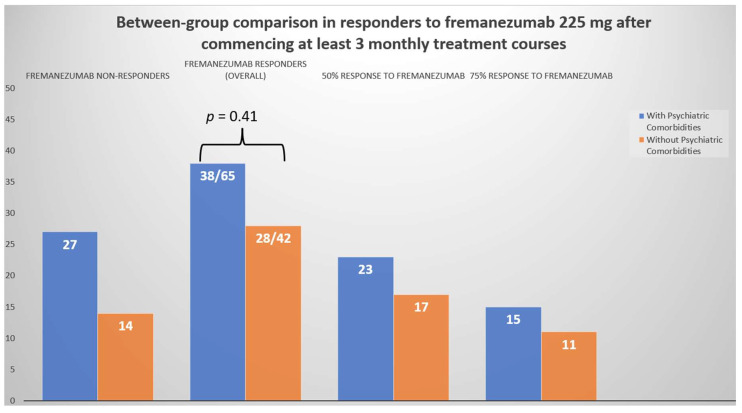
Differences in the number of fremanezumab responders at the last follow-up compared with baseline MHDs between patients with vs. without PCs.

Likewise, as depicted in Figure 2, the number of treatment responders with a ≥50% reduction in MHDs with fremanezumab was similar (*p* = 0.41) between patients with (*n* = 38/65; 58.5%) and without (*n* = 28/42; 66.6%) PCs.

## 4. Discussion

The current post hoc analysis sought to prospectively assess the efficacy of fremanezumab in CM patients with and without PCs in order to guide better treatment decisions by providing real-world evidence of outcomes with fremanezumab treatment in a homogenous cohort of CM patients with and without PCs, mainly anxiety and/or depression. Our main finding to emerge was that both CM patients with and without PCs comparably benefited from fremanezumab with significant reductions in MHDs and disability and an improvement in their QOL, while improvements in the severities of baseline PCs can also be anticipated during the course of treatment in about 45% of patients.

Our results are in agreement with previous findings from real-world studies demonstrating that fremanezumab was able to exert sustained reductions in MHDs across subgroups of migraine patients with comorbid anxiety and/or depression, whereas improvements in the severities of PCs might also occur in up to 50% of treated patients [27], with reductions in the number of patients who were prescribed antidepressants or anxiolytic medications [28]. These real-world data taken together, including ours, are supportive of the findings from post hoc analyses of data from the HALO CM study demonstrating that CM patients with comorbid depression and a baseline PHQ-9 score of 10 to 19, consistent with moderate-to-severe depression, experienced reductions in PHQ-9 of 9.5 to 10.5 points on average over the 12 weeks of the study when treated with fremanezumab [14]. Moreover, the results of a post hoc analysis pooling the results of two phase-three EM studies on the efficacy of another anti-CGRP MAb, i.e., galcanezumab, targeting the CGRP ligand (same as fremanezumab) for the prevention of migraine in patients with and without comorbid anxiety and/or depression showed that a comorbid medical history of anxiety and/or depression at baseline does not seem to interfere with the response to galcanezumab, and patients comparably respond regardless of their psychiatric history [29].

A possible shared pathogenesis of migraine and PCs with distinct pathophysiological mechanisms, overlapping or interacting with each other, has been previously suggested. However, it remains unclear whether migraine is caused by or is the cause of PCs. Hence, anxiety and/or depression may be able to trigger migraine attacks, while patients with frequent and severe MHDs, such as those suffering from CM, have increased susceptibility to be psychologically distressed. Shared genetic factors with evidence of a significant genetic overlap in identified loci (three SNPs: rs146377178, rs672931, and rs11858956 and two genes: ANKDD1B and KCNK5) for migraine and major depressive disorder might also cause the response in the bidirectional association between these medical conditions [30,31].

Nonetheless, neurochemical alterations—consisting of altered endocannabinoid and serum serotonin levels, which increase during migraine attacks and decrease in between them [32,33,34], coupled with decreased GABA cerebrospinal fluid levels in CM patients with comorbid depression [35]—may play a key role in the pathophysiology of such a psychiatric bidirectional comorbidity with migraine. Specifically, this serotoninergic imbalance has been suggested to alter the activity of the brainstem nuclei; enhance the activation of the trigeminovascular nociceptive pathway; and possibly also facilitate the generation of cortical spreading depression [36]. In accordance with the latter theory, medications acting on the serotoninergic system, including tricyclic antidepressants, and selective serotonin noradrenaline reuptake inhibitors are prescribed to patients for both EM and CM prophylaxis [37]. Moreover, dysregulation (hyperactivation) of the hypothalamic–pituitary–adrenal axis; estrogen and progesterone influence affecting the vascular endothelium and pain processing systems; neuroinflammation with elevated serum levels of inflammatory markers; and psychological factors, such as stress and sleep deprivation have been suggested to be involved in the intrinsic relationships between migraine and PCs [38,39,40]. Shared environmental factors and obesity might also contribute to the development of both migraine and comorbid depression and/or anxiety [41].

Finally, CGRP receptors are also implicated in the neurochemical alterations underlying a shared pathophysiology between migraine and PCs via their activation in the bed nucleus of the stria terminalis, strongly suggesting that the inhibition of CGRP receptors with the use of anti-CGRP MAbs may be a clinically useful strategy to achieve likely reductions in both migraine and PC severities [42]. From a clinical point of view, the involvement of CGRP in the pathophysiology of generalized anxiety disorder and possibly in depression might explain the dual beneficial effect of fremanezumab in reducing the severities of both migraine and PCs, as thoroughly demonstrated in this study.

It should be noted that our study, being a post hoc analysis, was not specifically designed to compare the two groups, namely, those with and without PCs, and this should be acknowledged as a limitation preventing the generalizability of our findings. In addition, our sample size was not very large, and this fact should also be noted as a limitation of the study. Nonetheless, our study included a homogenous sample of 107 fremanezumab-treated CM patients with difficult-to-treat migraine, who were studied with quantitative efficacy outcomes and with qualitative patient-reported tools for disability and QOL, and this thorough assessment should be counted among its strengths. Lastly, HADS represents an internationally acceptable instrument for rating psychological morbidity in migraine patients [43,44,45].

## 5. Conclusions

To conclude from a clinical point of view, our results indicate that fremanezumab appears to be effective as a preventive treatment in difficult-to-treat CM patients with or without PCs while also being beneficial in reducing the severity of comorbid anxiety and/or depression. The demonstrated efficacy, favorable safety, and tolerability of fremanezumab indicate its substantial therapeutic potential for patients with CM and comorbid PCs. Further studies specifically designed to compare PC subgroups and assess the changes in the severities of psychiatric symptoms over time with fremanezumab treatment are warranted. Towards meeting the latter need, the results of the ongoing UNITE study are anticipated [46] to further shed light on this clinically important issue.

## Figures and Tables

**Table 1:** Demographic and clinical characteristics of fremanezumab-treated CM patients with and without PCs.

Variable Participants *n* = 107	Patients without PCs *n* = 42 *n* %	Patients with PCs *n* = 65 *n %*
**Gender**		
Females	34 80.9	59 90.8
Males	8 19.1	6 9.2
**Age ± SD (range)**	49.6 ± 10.1 (26–70)	50.1 ± 11.1 (23–70)
**Number of previously used preventative medications**		
Median value (range)	5 (3–7)	5 (3–8)
**Years ± SD (range) with chronic migraine**	29.8 ± 9.9 (12–41)	28.0 ± 9.6 (7–45)
**Body mass index status**		
Normal (18–24.9)	22 52.4	37 56.9
Overweight (25–29.9)	17 40.5	18 27.7
Obese (>30)	3 7.1	10 15.4
**Psychiatric comorbidities**	0 0	65 100
Anxiety disorder	0	24
Depression	0	13
Mixed anxiety and depression disorder	0	26
Bipolar disorder (stable—in remission)	0	2
**Medication-overuse headache**		
Yes	37 88.1	61 93.8
No	5 11.9	4 6.2

## Data Availability

The data that support the findings of this study are available from the corresponding author upon reasonable request.
